# Novel *in vivo *targets of ΔNp63 in keratinocytes identified by a modified chromatin immunoprecipitation approach

**DOI:** 10.1186/1471-2199-8-43

**Published:** 2007-05-23

**Authors:** Barbara Birkaya, Kori Ortt, Satrajit Sinha

**Affiliations:** 1 Department of Biochemistry, State University of New York at Buffalo, Buffalo, NY 14214, USA

## Abstract

**Background:**

p63 is a transcription factor that plays an important role in skin epidermal development and differentiation. The p63 gene encodes for two major protein isoforms, those containing an amino-terminal trans-activation domain (TAp63) and those lacking this domain (ΔNp63). Both the TA and ΔN transcripts are also alternatively spliced at the 3' end producing proteins with unique C-termini that are designated as α, β and γ isoforms. Recent research has suggested that ΔNp63 is the predominant isoform expressed and active in keratinocytes.

**Results:**

To better elucidate the biological role of p63 in regulating gene expression in keratinocytes we performed chromatin immunoprecipitation (ChIP) experiments with ΔNp63-specific antibodies. We included an additional step in the ChIP procedure to enrich for ΔNp63 targets by screening the library of immunoprecipitated DNA for its ability to bind recombinant GST-ΔNp63. Cloning of ΔNp63-ChIP-derived DNA fragments identified more than 60 potential ΔNp63 target loci that were located close to or embedded within known or predicted genes. Identity of these target genes suggests that they may participate in a myriad of cellular processes including transcriptional regulation, signaling and metabolism. Here we confirm the binding of ΔNp63 to several of these genomic loci both by EMSA and replicate ChIP assays. Finally we show that the expression of many of these target genes is altered when ΔNp63 levels in keratinocytes are reduced by siRNA, further confirming that these are bona fide targets.

**Conclusion:**

This unbiased genomic approach has allowed us to uncover functional targets of ΔNp63 and serves as the initial step in further analysis of the transcriptional regulatory mechanisms that are governed by p63 in keratinocytes.

## Background

The transcription factor p63, which belongs to a family of genes that also include p53 and p73, plays an important role in the transcriptional regulation of many biological processes including development, differentiation and apoptosis[1,2]. Interestingly, p63 exhibits a restricted spatio-temporal expression pattern with high levels reported in epithelial cells. Indeed, the function of p63 has been predominantly examined in stratified epithelium in many organs such as the skin, mammary glands, prostate etc. Both gain-of-function and loss-of-function studies have clearly demonstrated that p63 is a critical master regulator of the epithelial differentiation program[1,3]. This is quite evident in the dramatic phenotype of the p63 knockout mice, which lack stratified epithelium and their derivatives in multiple tissues and organs[4,5].

The biological function of p63 is mediated by several isoforms derived from distinct transcripts that are generated from a complex genomic structure. The *p63 *gene gives rise to two major transcript variants through the use of distinct promoters, which are located far apart from each other[6]. The proximal promoter located upstream of exon1 directs the expression of transcripts that encode for an amino terminal transactivation domain (TA), whereas an internal promoter embedded within the third intron controls the expression of transcripts that lack this domain (ΔN). In addition, both the TA and ΔN transcripts are differentially spliced at the 3' end to generate proteins with unique C-termini that are designated as α, β and γ isoforms of p63.

All isoforms of p63 share a DNA-binding and an oligomerization domain, which shows sequence conservation with p53. Hence, these proteins are capable of sequence specific DNA-binding to p53 response elements and related sequences. Although the ΔNp63 isoforms were initially thought to function by exerting dominant negative effects on TAp63, it is increasingly becoming clear that the ΔNp63 proteins also mediate direct transcriptional activation and repressor activities on target genes [7-10]. In view of the fact that there are high levels of ΔNp63 protein but not TAp63 in many epithelial cells and that the ΔNp63 isoform is the only form of p63 present in lower organisms, it is thought that ΔNp63 may be the primary mediator of the biological function of the *p63 *gene.

Since p63 is a transcription factor, it is likely that it governs the various cellular processes and developmental decisions by regulating specific target genes. Although p63 has been shown to regulate some well-characterized p53 responsive genes, it is becoming increasingly evident that there exists a unique set of p63 target genes[11]. This notion is further strengthened by the fact that p63 has a distinct functional role in development and that the DNA-binding activity of these two proteins exhibits clear differences[1,12,13]. It is also possible that both p53 and p63 can regulate common target genes as exemplified by maspin, IGFBP-3 and PERP, which though initially were thought to be regulated by p53 are now clearly proven to also be p63 targets [14-17]. To identify additional p63 targets, some laboratories have utilized an experimental model system where cells lacking p63, such as Saos2 or HEK 293, were assessed for global alterations in gene expression by microarray analysis under conditions where p63 is over expressed[12,18,19]. Although these and similar other studies have unearthed some interesting potential p63 targets, identifying the exact location of the cis-elements that mediate the transcriptional effects of p63 have remained elusive[20]. Hence, a direct examination of p63-responsive elements in epithelial cells, which express high levels of p63 under physiological conditions, is warranted.

Keratinocytes express high levels of ΔNp63 and have provided a useful model system to dissect the role of p63 and to study its target genes[10]. These studies have been facilitated by chromatin immunoprecipitation (ChIP) experiments to confirm known p63 binding sites within the promoters of selected downstream targets *in vivo*. Although ChIP can be also a powerful tool to identify previously unknown targets of transcription factors, technical limitations have often precluded such an experimental strategy. In this paper, we report our initial results with an improved genomic ChIP approach to isolate and characterize 62 potential genomic ΔNp63 binding sites in keratinocytes. These sites are associated with various genes involved in a wide variety of cellular processes including transcription, signaling and metabolism. As proof of principle we have confirmed the association of ΔNp63 with a subset of these targets both *in vitro *and *in vivo*. Finally, we demonstrate that the expression levels of the potential ΔNp63 target genes located close to these genomic binding locations are affected when p63 levels are lowered. Collectively, our studies highlight the robustness of our experimental strategy to unearth novel targets for ΔNp63 and thus can be extended to other transcription factors.

## Results

### A modified ChIP strategy for enrichment and cloning of ΔNp63-target elements

**Figure 1:**
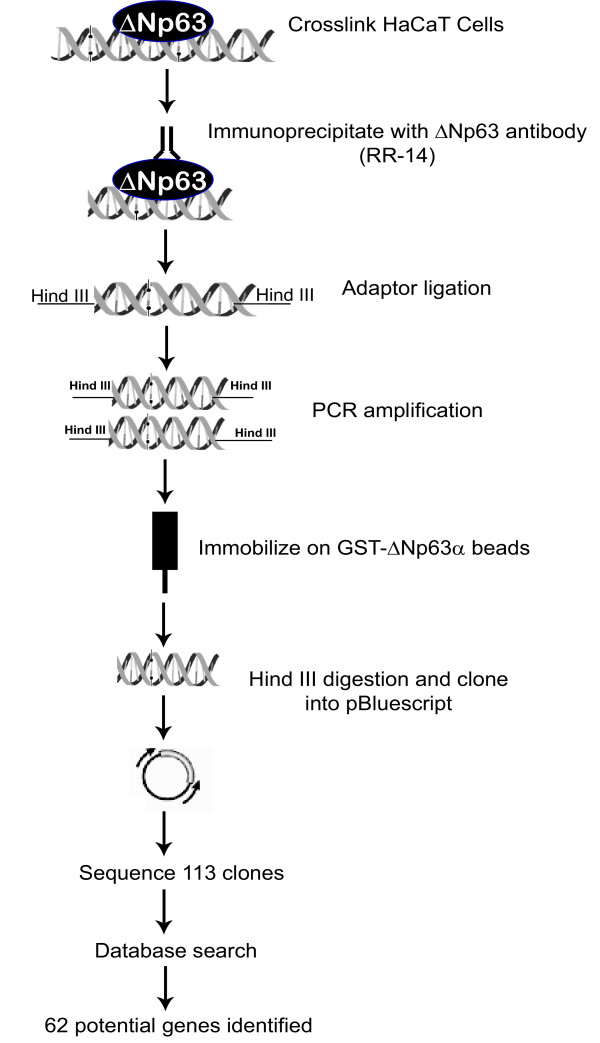
**A schematic representation of the modified chromatin immunoprecipitation approach used to identify potential target genes of ΔNp63**. Following chromatin immunoprecipitation, to enrich for ΔNp63 targets, ChIP products were ligated to linkers and amplified by PCR using the linker sequence for priming. The PCR-amplified products were subsequently incubated with agarose beads containing GST-ΔNp63 protein. ChIPed fragments were then digested with Hind III, cloned, and sequenced.

To better understand the mechanisms by which ΔNp63 transcriptionally regulates the keratinocyte development and differentiation program we sought to identify its *in vivo *target genes by a ChIP strategy. For this purpose we utilized HaCaT cells, which are immortalized human keratinocytes that have been widely used to study keratinocyte biology. These cells express high levels of ΔNp63, which can be detected by an isoform-specific antibody RR-14 that has been generated by our laboratory and has been successfully used in ChIP experiments[21]. ChIP strategy is widely utilized to examine if a specific genomic site is occupied by a transcription factor in the context of a living cell. However, identification of unknown targets by cloning and sequencing the DNA that is obtained from the immunoprecipitated material has proven to be technically challenging. This is mostly due to limiting amounts of DNA obtained during the procedure and overwhelming excess of non-specifically precipitated DNA. To overcome these issues, we incorporated two additional steps in our experimental scheme to improve the efficiency of obtaining ΔNp63 targets. First we utilized a ligation-mediated PCR technique, during which the ChIP products obtained after immmunoprecipitation were ligated to linkers and amplified by PCR using the linker sequence for priming[22]. This allowed us to obtain sufficient material for subsequent purification and cloning (Figure 1). Second, to reduce the contamination with non-specific DNA fragments, we purified the PCR-amplified fragments by incubation with agarose beads containing GST-ΔNp63α protein. This enrichment procedure allowed us to select for DNA fragments that are more likely to contain binding-sites for ΔNp63. The DNA fragments obtained from chromatin immunoprecipitation were then digested with restriction enzyme (present in the linker) and cloned into pBluescript vector as described in Materials and Methods. We isolated and sequenced 113 individual clones that represented the DNA fragments immunoprecipitated by ΔNp63.

### The location and identity of the ΔNp63 target genomic DNA fragments obtained after ChIP cloning

We searched the human genomic database by BLAST to determine the location of the immunoprecipitated DNA fragments in relation to known or predicted genes. This analysis revealed that 62 of the extracted DNA fragments immunoprecipitated by ΔNp63 mapped in proximity to known, annotated or predicted genes (those supported by mRNA sequences, or predicted by expressed sequence tags, EST). We used the following criteria to assign the DNA fragments to a specific gene: if the sequence matched a) to the intragenic region of a known or predicted gene or b) to a region within 100 kb upstream or downstream of a gene. Tables 1, 2, 3 list the genomic distribution of the 62 gene-associated ΔNp63 binding fragments. The remaining ChIP fragments that were obtained from sequencing were not analyzed further since they either corresponded to non-annotated regions of the human genome or no mRNA or EST sequence that would predict a gene was found within a 100 kb distance of the DNA fragment. It is possible that some of these genomic sequences may represent distal enhancers involved in regulating gene expression from distances significantly farther than 100 kb as has been demonstrated for many enhancers. Alternatively, these segments may denote non-annotated regions of the genome that do not encode for conventional genes but are sites for miRNA and similar elements. Some of these elements may also represent experimental artifacts resulting from non-specific DNA-binding of ΔNp63 to certain chromatin regions that are captured during formaldehyde cross-linking or contaminating DNA obtained during the immunoprecipitation or PCR enrichment steps.

**Figure 2:**
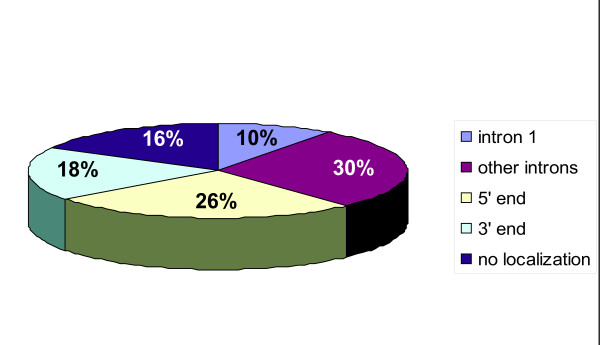
**Location of the identified p63 ChIPed DNA sites relative to known gene**. Examination of 60 potential target genes demonstrates that p63 binding sites are located within introns as well as at the 5' end and 3' end of these target genes.

Examination of the sixty-two potential ΔNp63 target genes revealed several interesting findings. Forty percent of the ΔNp63-ChIP fragments were located within an intron of known or predicted genes, with a quarter of them in the first intron (Tables 1, 2, 3 and figure 2). This observation is in agreement with many other transcription factors such as Myc and Sp1 for which genomic binding sites have been deciphered on a large scale and reflects the growing evidence for the presence of intragenic *cis*-regulatory regions for transcriptional control. A further forty-four percent of the DNA fragments chromatin immunoprecipitated by ΔNp63 were located within the region spanning 100-kb upstream or downstream of candidate target genes. Although several sites were located less than 10-kb upstream of a transcription start site, only a small number mapped to the promoter proximal regions. This may reflect the propensity of ΔNp63 to act primarily through distal enhancers or simply signify under-representation of promoter regions since they are not well defined and properly annotated for a vast majority of human genes. Interestingly, for a distinct number of cases the sequence chromatin immunoprecipitated by ΔNp63 corresponded to a region that was close to two genes; for example fragment A8 mapped to a segment that was at the 5' end of the *NAPA *gene but also closely located at the 3' of *ZNF541 *gene (Table 3). In such situations, the close proximity of the p63-response element to both of these potential target genes makes it difficult to ascertain whether either one or both of these genes are bona-fide ΔNp63 targets without further experimental evidence. Functional classification of these potential targets based on Gene Ontology categorization revealed that these are widely distributed among a wide variety of categories including transcriptional regulation, signaling cascades and metabolism. Of note some of the potential ΔNp63 target genes that we identified in our screen have been previously shown to be regulated by p63 (Table 1 and 3). These data reaffirms the validity of our ChIP based cloning approach and offer further evidence that some of these previously identified targets of ΔNp63 are direct ΔNp63 transcriptional targets and more importantly identifies the location of the ΔNp63-response elements that might be responsible for such an effect.

### Expression of a subset of ΔNp63 target genes in HaCaT cells and confirmation of ΔNp63 binding by independent ChIP assays

**Figure 3:**
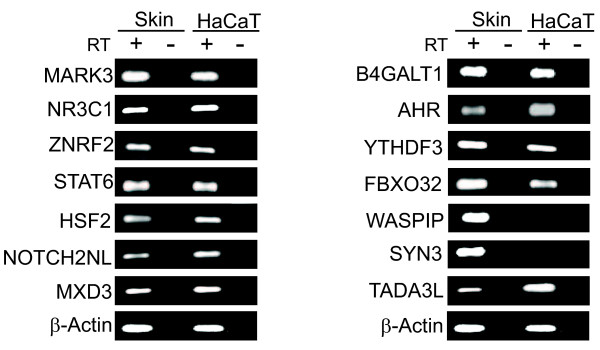
**Expression of ΔNp63 target genes in human skin and HaCaT cells**. Total RNA was isolated from human skin and HaCaT cells and RT-PCR was performed using the primers listed in Additional File 3A to specifically amplify the target genes. β-Actin serves as a control.

**Figure 4:**
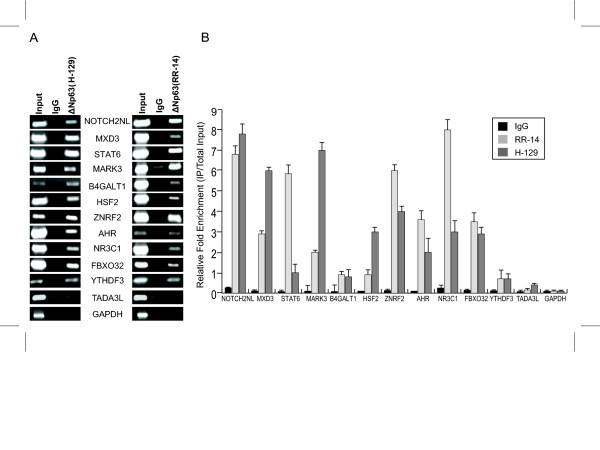
**Independent ChIP assay demonstrates binding of p63 to ChIPed DNA sequences**. ChIP was performed on HaCaT cells with N-terminal RR-14 and C-terminal H-129 anti-p63 antibodies. (A) Enrichment of target genes relative to IgG on an agarose gel. (B) Real-time PCR demonstrating the relative enrichment of p63 target genes with p63 antibodies and IgG. Primer sequences utilized are listed in Additional File 3B.

To facilitate further studies of the potential ΔNp63 targets, we selected fourteen loci from the sixty initially identified by ChIP. These DNA fragments immunoprecipitated by ΔNp63 were arbitrarily chosen and represented different locations relative to the target genes and genes that were involved in a variety of cellular processes. For simplicity we focused mostly on ΔNp63 sites that were located close to a single gene. First we performed RT-PCR experiments to test if these genes were indeed expressed in HaCaT cells. As a control we used human skin. Of the fourteen candidates chosen, two of them *WASPIP *and *SYN3 *showed no detectable expression in HaCaT cells (Figure 3). It is possible that these two genes are not expressed in HaCaT cells in the specific cell culture conditions used or that their normal expression is repressed by p63. Alternatively, expression of these genes may be restricted to the dermal or other non-keratinocyte compartment of the skin. The remaining twelve genes showed expression in both HaCaT cells and in skin and were chosen for further studies. Since these twelve genes are co-expressed with ΔNp63 in HaCaT cells, we next confirmed the binding of ΔNp63 to the isolated ChIP fragments by performing independent ChIP assays (Figure 4). For these experiments, cross-linked chromatin from HaCaT cells was immunoprecipitated in independent experiments with two different anti-p63 antibodies and the co-precipitation of the ΔNp63-response elements was ascertained by PCR. The antibodies we chose were an N-terminal specific antibody, RR-14, which was utilized in the original genomic screen and a commercially available anti-p63 antibody, H-129 that recognizes the C-terminal domain of the ΔNp63α protein. We utilized sets of primers that amplify the twelve fragments immunoprecipitated by ΔNp63 and as a negative control we used a set of primers that amplify a genomic segment in the *GAPDH *gene. As shown in Figure 4A, after immunoprecipitation of cross-linked chromatin we found that there was specific enrichment of eleven potential ΔNp63-response elements with both antibodies against p63 compared to IgG control. In contrast, we did not observe any localization of ΔNp63 to the *GAPDH *or *TADA3L *genomic loci as demonstrated by the negative PCR results. Similar results were obtained from three independent ChIP experiments. The relative enrichment of each fragment was also examined by quantitative PCR in at least three independent ChIP experiments (Figure 4B). For this, we performed parallel ChIP assays where either the two anti-p63 antibodies or IgG was used for immunoprecipitation. The relative fold enrichment of ΔNp63-ChIP fragments was determined by comparing the amount of fragment-specific PCR product amplified from the anti-p63 ChIP, and the negative control (IgG) ChIP samples. For eleven of the ΔNp63-ChIP fragments examined significant enrichment was observed with anti-p63 antibody, except TADA3L and control GAPDH. This specific enrichment suggests that the DNA fragments chromatin immunoprecipitated from the genomic screen represent *bona fide *binding sites for ΔNp63 in HaCaT cells. The fact that the *TADA3L *locus could not be amplified under the replicative ChIP conditions suggests that this may be a false positive.

### Direct binding of ΔNp63 to p63-response elements located within the DNA fragments immunoprecipitated by ΔNp63

**Figure 5:**
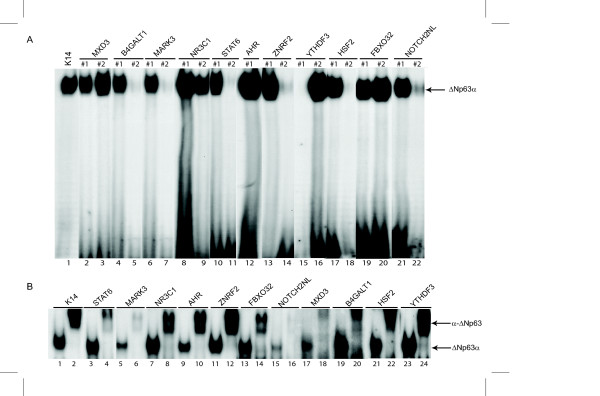
**EMSA demonstrates binding of ΔNp63 to p63 binding sites located within the ChIPed DNA sequences**. (A) EMSAs were performed by radiolabeling oligonucleotide probes containing p63 binding sites from the various ChIPed DNA sequences corresponding to known genes as indicated above each lane. Two radiolabeled oligonucleotide probes from each ChIPed sequence were each incubated with recombinant His-tagged ΔNp63α protein. Arrow indicates ΔNp63α. (B) EMSA was performed with nuclear extract from HaCaT cells and incubated with the radiolabeled oligonucleotide probe that bound best to recombinant protein demonstrated in A above. A complex containing ΔNp63 is supershifted with an anti-ΔNp63 antibody. See Additional File 1 for oligonucleotide sequences used in EMSA.

Of the fourteen potential ΔNp63 targets, eleven were expressed in HaCaT cells and independent ChIP experiments suggested that ΔNp63 could associate with a genomic fragment present within or close to these genes. Since this association of ΔNp63 to such sites could be either direct or indirect, we analyzed the eleven DNA fragments for the presence of p63 consensus DNA binding sites. Our recent experiments have defined the optimal p63 DNA-binding consensus motif as (T/A)A(T)ACA(T)TGT(T/A)T consisting of a CA(T)TG core and AT-rich 5' and 3' flanking sequences[13]. Indeed many of the known p63 target genes contain such a p63-response element in their regulatory regions. We found that the eleven DNA fragments chromatin immunoprecipitated contained at least one stretch of sequence that matched closely the p63-consensus, and in several cases more than one potential p63-response element was detected. To experimentally test these p63-like response elements, we designed oligonucleotide probes (see Additional File 1 for sequences) for two of the sites from each locus that most closely matched the p63-consensus and performed Electrophoretic Mobility Shift Assays (EMSA). As a control, we utilized a p63-response element from the K14 enhancer that has been demonstrated to be a bona-fide p63 binding site[10]. We used similar molar amounts of each radiolabeled oligonucleotide to allow the relative binding to each probe to be compared. EMSA showed that recombinant ΔNp63 was capable of binding strongly to at least one of the p63-response elements present in the immunoprecipitated DNA fragments corresponding to all eleven target genes (Figure 5A). For some target genes, such as *MXD3*, *NR3C1 *and *FBXO32 *both the p63-response elements showed similar binding strength to ΔNp63. However, for the remaining targets only one of the oligonucleotide probes bound to ΔNp63 suggesting that flanking sequences likely influence the DNA-binding specificity as shown previously by our laboratory[13].

Having shown that these oligonucleotides containing p63-response elements can bind to recombinant ΔNp63 purified from bacteria, we wanted to test if they could also bind to native ΔNp63 present in cells. For this purpose we utilized nuclear extracts from HaCaT cells and repeated EMSAs with the oligonucleotide that demonstrated the best binding to recombinant ΔNp63 for each of the eleven target genes. Upon incubation with labeled oligonucleotide, a specific complex was observed with HaCaT nuclear extracts in each case. This complex was clearly formed by ΔNp63, since addition of anti-p63 antibodies resulted in a distinct supershift (Figure 5B). Taken together our EMSA studies suggest that the fragments immunoprecipitated contain legitimate p63-response elements that can bind to recombinant ΔNp63 and more importantly to endogenous ΔNp63 present in keratinocytes.

### Transcriptional activation of the DNA fragments chromatin immunoprecipitated by ΔNp63

**Figure 6:**
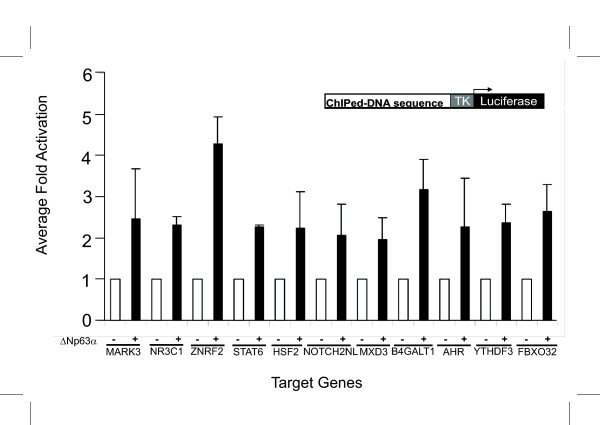
**ΔNp63α activates the ChIPed DNA sequences in reporter gene assays**. Luciferase constructs containing the various ChIPed DNA sequences cloned upstream of a heterologous TK promoter demonstrate high levels of activity when co-transfected with an expression plasmid encoding ΔNp63α in PtK2 cells. Luciferase values were determined and normalized against β-galactosidase values. Corrected luciferase values for cells transfected with empty vector pCMV-HA were set at 1. TK; thymidine kinase

Having shown that ΔNp63 can bind to these eleven ChIPed segments both *in vitro *and *in vivo *we next examined their responsiveness to ΔNp63 in transient transfection experiments. For these experiments we chose PtK2 cells since these have been shown to lack any endogenous p63 expression. First we cloned each of the eleven fragments corresponding to the genomic regions containing p63-response elements in a luciferase reporter plasmid upstream of the heterologous thymidine kinase (TK) promoter. Each reporter plasmid was co-transfected with either an expression plasmid encoding for HA-tagged ΔNp63α or an empty HA control vector. As shown in Figure 6, expression of ΔNp63α resulted in increased levels of reporter activity (2–5 fold) for each of the eleven constructs compared to empty vector. These data along with those obtained from the previous ChIP experiments and EMSA strongly argue that ΔNp63 activates these eleven target genes through the p63-response elements.

### Inhibition of p63 in HaCaT cells affects the expression of ΔNp63 target genes

**Figure 7:**
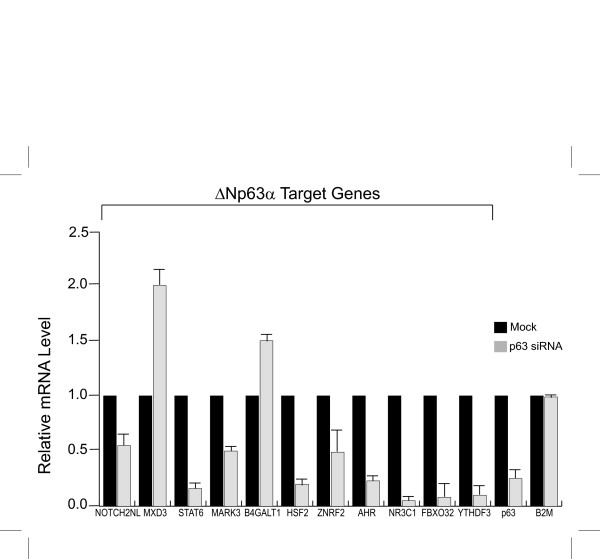
**Expression of target genes is altered when p63 expression is knocked down by siRNA**. HaCaT cells were transfected with p63 siRNA. Expression of target genes was analyzed by Real-time PCR. Mock-transfected cells were used as a control. Primer sequences used are listed in Additional File 3A.

The fact that ΔNp63 can bind and activate the regulatory elements associated with the target genes suggests that ΔNp63 might be a transcriptional regulator for these genes. Hence, to demonstrate more directly whether ΔNp63 is involved in regulating expression of the putative ΔNp63 target genes, we examined the effect of inhibiting p63 expression in HaCaT cells. We used synthetic dsRNA (siRNA) to reduce p63 expression in HaCaT cells. As shown in Figure 7, the use of siRNA led to a significant reduction in the expression of p63 mRNA levels in cells transfected with the p63 siRNA. This reduction in p63 amounts was also evident at the protein level (Additional File 2). We performed at least three quantitative RT-PCR assays using cDNA synthesized from three independent RNAi experiments to examine the expression of the eleven putative p63 target genes. The housekeeping gene *B2M *(beta2 microglobulin) was used as a reference standard. As shown in Figure 7, expression of six of these genes *NR3C1*, *STAT6*, *HSF2*, *AHR*, *YTHDF3*, *and FBXO32 *were dramatically reduced by more than 75% in response to p63 siRNA. This indicates that a threshold level of p63 is required to maintain expression of these genes. On the other hand, three target genes *ZNRF2*, *NOTCH2NL*, and *MARK3 *showed more modest reduction in mRNA levels of approximately 50%. This relatively modest reduction in expression of these target genes may be due to the fact that persistent low level of p63 is sufficient to maintain their expression. Alternatively, regulation of these target genes may involve additional repressors and/or activators that may compensate for the lack of p63. Interestingly, two genes, *MXD3 *and *B4GALT1 *were modestly up regulated when p63 levels were low, suggesting that p63 might play a role in transcriptional repression of these two targets. This is despite the fact that in transient transfection experiments, the DNA fragments immunoprecipitated by ΔNp63 corresponding to both *MXD3 *and *B4GALT1 *were positively activated by ΔNp63 (Figure 6). This implies that the transcriptional regulation process of these two potential targets *in vivo *is more complex and that either additional direct p63-response elements exist or indirect effects from knockdown of p63 might influence the balance of positive and negative transcriptional regulators. Overall, our data suggests that of the subset of genes identified by ΔNp63-ChIP and examined in this study, a large proportion of them are likely to be genuine regulatory targets of ΔNp63 in HaCaT cells.

## Discussion and conclusions

Identifying target genes is critical in understanding the mechanism by which the transcription factor p63 regulates the intricate process of epithelial development and differentiation. Towards this end, several laboratories have undertaken a target discovery approach that involves manipulating the activity of p63 followed by gene expression analyses[12,18,23,24]. Although such genome-wide microarray analysis has been valuable and has led to the identification of several transcripts that are potentially turned on and off by p63, these experiments have several limitations. First, it is difficult to ascertain whether a responsive gene is a direct or an indirect target of p63. Second, in experiments based on over expression of p63, it is likely that the response of some genes is driven by the exaggerated non-physiological concentration of this transcription factor. Third, the selected cell types that have often been utilized for these studies do not express endogenous p63 and thus may not represent the normal cellular milieu under which p63 operates. Finally, such studies a priori do not provide any information on the location of the p63-responsive elements that control the expression of target genes. Search for such regulatory elements is further hampered by the degenerate nature of the p63 DNA-binding sequence. Indeed, bioinformatics approaches often identify an unrealistically large number of potential p63 binding sites without distinguishing those that are functionally relevant. Hence, despite the identification of many p63 target genes, it has been difficult to ascertain whether p63 is involved in direct regulation of those genes by binding to their promoter and/or enhancer elements.

In contrast, here we have undertaken a direct approach of ChIP in combination with sequencing and mapping of isolated genomic DNAs to locate binding sites for ΔNp63 and its potential target genes *in vivo*. Indeed, such ChIP strategies have been successfully utilized to not only determine whether a candidate genomic site is occupied by a specific transcription factor but to also identify transcription factor targets. However, in such cases it has been technically challenging to separate out the specific regulatory DNA segment that is bound to the transcription factor from the vast excess of non-specifically precipitated DNA. To overcome these challenges, we have fine-tuned the ChIP procedure and modified it to successfully identify *in vivo *ΔNp63 target genes from HaCaT cells, which express high levels of ΔNp63, the major isoform of p63 protein in keratinocytes. First, since the immunoprecipitated DNA is of limited amount, we utilized a ligation-mediated PCR technique, in which the ChIP products were ligated to linkers and amplified by PCR using the linker sequence. This allowed us to obtain sufficient material for subsequent purification and cloning. Second, to eliminate non-specific DNA fragments prior to cloning the ChIPed DNA, we purified the PCR-amplified fragments by incubating them with agarose beads containing GST-ΔNp63α. This step ensures that fragments that contain DNA binding-sites for ΔNp63 are selectively enriched and can be separated from non-specific DNA. The potential caveats to such a purification step are that some DNA fragments containing genuine ΔNp63-targets might fail to bind to recombinant GST-ΔNp63α or that non-specifically precipitated DNA fragments can fortuitously bind to GST-ΔNp63α under the *in vitro *conditions of the assay. In addition, genomic segments to which ΔNp63 are recruited indirectly for example through protein-protein interactions are likely to be under-represented in this experimental scheme. However, our results suggest that the benefits of utilizing such an enrichment step clearly outweigh the potential drawbacks of the overwhelming background of non-specific genomic immunoprecipitated DNA.

One of the interesting findings that emerge from our studies is that the vast majority of these ΔNp63-binding sites are not localized to the proximal promoters of the target genes. This is in agreement with recent genome-wide target identification studies on other transcription factors such as Sp1, p53 and Myc, which demonstrate that a majority of the binding sites are not in the proximal promoter but rather scattered[25]. Interestingly, a large subset of the ΔNp63 targets were not close to any known gene; these could represent potential long range enhancers or unknown genes that have not been annotated or non-coding RNAs. Taken together, our study reinforces the need to not limit examination of the proximal promoter region only when searching for direct targets of transcription factors.

Our improved ChIP-based screening strategy allowed us to identify a large number of ΔNp63-response elements and their corresponding targets. Here we have examined a subset of the ΔNp63 target genes in detail. Our data show that the majority of the candidate target genes are co-expressed with ΔNp63 in HaCaT cells and that the specific segment isolated from the cloning of DNA immunoprecipitated is indeed occupied by ΔNp63 as determined by independent ChIP experiments. We also demonstrate that these segments harbor at least one p63 response element that can bind to both recombinant ΔNp63 and ΔNp63 present in HaCaT nuclear extract and that these segments can be transcriptionally activated by ΔNp63α in transient transfection assays. Finally, reduction in the levels of ΔNp63 in HaCaT cells led to significant decrease in transcript levels for a majority of the p63 target further validating the relevance of p63 in regulating these genes and confirming that the eleven genes fulfill the criteria to be direct transcriptional targets of p63. Only two genes, *MXD3 *and *B4GALT1 *were de-repressed even though in transient transfection experiments the DNA fragments immunoprecipitated by ΔNp63 corresponding to both *MXD3 *and *B4GALT1 *were positively activated. The fact that the majority of the targets identified by our approach are activated by ΔNp63 may reflect a bias towards selection of a high affinity p63-binding site due to the incorporation of an additional purification step introduced in the cloning procedure. However it is clear from numerous studies that ΔNp63 also represses transcription (as evident by our results for *MXD3 *and *B4GALT1*). This might potentially be mediated through interaction of ΔNp63 with non canonical response sites. Collectively, our data support the growing consensus that the ΔNp63 isoform is primarily involved in transcriptional activation of target genes rather than merely acting as a dominant negative that opposes the function of TAp63.

The diverse nature of the targets identified in our study reinforces the notion that p63 plays a role in complex biological pathways that affect a wide variety of cellular processes. As a critical regulator of keratinocyte development and differentiation, it is not difficult to imagine p63 as a focal point in the transcriptional network and cascades. This role of p63 as master regulator is supported by a large number of potential targets identified in this study that are transcription factors such as MXD3 and STAT6. Similarly our identification of *NOTCH2NL *as a ΔNp63 target gene contributes to the growing list of Notch family members and effectors of this pathway that are regulated by p63[19,26]. The p63 targets uncovered in our study include genes previously linked to p63, such as *DDR1*. Discoidin domain receptor 1 (DDR1) is a unique receptor tyrosine kinase activated by various types of collagens and is known to play a role in epithelial cell attachment, migration, survival, and proliferation[27,28]. Upregulation of DDR1 by p63 has been observed in several cases by gain of function studies; our ChIP analysis now clearly demonstrates that DDR1 is a direct transcriptional target[12,29]. What is also clear from surveying the list of ΔNp63 targets is that there are predicted genes with unknown functions. Regulation of these unknown genes by ΔNp63 may be one underlying mechanism by which p63 may mediate some of its myriad biological activity.

As this manuscript was in preparation, two independent studies were published in which genome-wide p63 binding sites were determined by ChIP-Chip technology. In the first study, the Mantovani group performed ChIP-on-Chip experiments on HaCaT cells using two different platforms; the 12K CpG islands and 12K promoter arrays that led to the identification of ~200 target loci [30]. In the second study, Yang et al utilized genome wide tiled microarrays covering the entire nonrepetitive human genome, that lead to identification of ~5800 target sites for p63[31]. These studies were performed in ME180 cervical carcinoma cell line with the 4A4 anti-p63 antibody that recognizes all p63 isoforms. A closer look at these data and that of ours presented in this paper show that the data sets generated from these studies display significant overlap but also distinct patterns. Indeed twenty-five percent of the ΔNp63 target genes that we report in this work have also been shown to be putative p63 target genes based on these two studies (Table 1 and 3). The differences in the data sets probably result from experimental noise associated with the ChIP-ChIP experiments, differences in cell culture conditions such as cell type and/or proliferation state and the distinct antibodies utilized in these studies. The presence of different p63 isoforms and their unique properties surely adds to this complexity.

An interesting point to be considered is that for many of the p63 targets, it is likely that there are more than one p63-response elements. These elements are thus scattered in the proximal promoter, in the intragenic region and at a distance far from both the 5' and 3' end of the gene itself. Pertinent to this notion, we find a p63-response element in the third intron of the *DDR1 *gene, whereas the data from Yang et al., point to p63-response element located 5' of the gene [31]. It is likely that both these sites are bona-fide p63 binding sites. The presence of multiple p63-response elements reflects the importance of p63 in regulating DDR1 gene expression. This situation is similar to a well-characterized p63 target, the *K14 *gene, where p63 binding sites are present both in the proximal promoter and a distal enhancer[10]. Because each experimental condition captures only a snapshot of the entire p63 directed transcriptome, it is important that additional studies are performed to take into account the dynamic cellular environment in which p63 operates. Our approach described in this paper validates the use of ChIP coupled with an enrichment strategy to identify transcriptional targets *in vivo *and demonstrates the feasibility of such an approach that can be applied on a large scale. The various complementary strategies should provide a starting point to dissect out the network of relevant p63 targets and offer a strong basis for the elucidation of the gene regulatory pathways that are controlled by p63 in keratinocytes and other cell types.

## Methods

### Cell culture

HaCaT cells were maintained in DMEM supplemented with 10% fetal bovine serum and 100 U/ml penicillin and 100 μg streptomycin. Cells were routinely passaged at 90% confluency. Ptk2 (rat kangaroo kidney epithelial) cells were grown in minimal essential medium supplemented with 10% fetal bovine serum, 1% MEM non-essential amino acid solution, 100 U/ml penicillin and 100 μg/ml streptomycin.

### Chromatin Immunoprecipitation (ChIP) assays and cloning of immunoprecipitated products

Chromatin immunoprecipitation experiments were performed using HaCaT cells with anti-p63 antibodies as described previously[21]. To facilitate cloning immunoprecipitated DNA was amplified by linker mediated PCR. Linker DNA consists of two annealed oligonucleotides: 5'-AGAAGCTTGAATTCGAGCAGTCAG-3', phosphorylated at the 5' end, and 5'-CTGCTCGAATTCAAGCTTCT-3', containing a Hind III restriction enzyme site. Linkers were ligated to the immunoprecipitated DNA with T4 DNA ligase (Invitrogen) and amplification was carried out directly, without purification of ligated DNA. Immunoprecipitated DNA after amplification was diluted 1:2 with DNA binding buffer (20 mM Tris pH 7.8, 50 mM NaCl, 1 mM MgCl_2_, 0.2 mM EDTA, 5% glycerol, 0.5 mM DTT, 0.5 mM PMSF) and incubated with Glutathione Sepharose 4B beads (Amersham Biosciences) loaded with ΔNp63α for 2 hours to enrich for p63 binding sites. DNA was eluted with buffer 1% SDS and 100 mM NaHCO_3 _and subsequently phenol-chloroform purified. Linker DNA was removed by digestion with Hind III restriction enzyme and purified with PCR purification kit (Qiagen) and cloned into pBluescript. Clones were sequenced and analyzed in silico.

### *In silico *data analysis

Clones were sequenced and a BLAST search of the human genome database at NCBI was performed to identify potential target genes. Genes located within 100 kb of the ChIPed-DNA sequence were considered as potential p63 targets. In situations where two genes were located within this range, the closest gene was chosen. In cases, where the sequence was within equal distance of two genes, both genes were chosen.

### RT-PCR

Total RNA from HaCaT cells was isolated and purified by TRizol (Invitrogen) according to established protocols. Two μg of total RNA from HaCaT cells and human skin (Stratagene) was reverse transcribed with the iScript cDNA synthesis kit (Bio-Rad). The primers listed in Additional File 3A were designed to span at least one intron. β-actin was used as a control and a 425 bp fragment was amplified with specific primers 5'-GCTCACCATGGATGATGATATCGC-3' and 5'-GATAGCATAGCCTGGATAGCAACG-3'. Jump Start Taq Polymerase (Sigma-Aldrich) was used for PCR amplifications.

### Confirmation of p63 binding to target sequences by ChIP

Selected p63 target sites were evaluated for binding by p63 by independent ChIP assays utilizing immunoprecipitated DNA from two different anti-p63 antibodies, H-129 (Santa Cruz) and RR-14 [10]. Primers utilized for ChIP assays are listed in Additional File 3B. Real time PCR conditions were similar to the one described for siRNA knockdown of p63.

### Preparation of recombinant p63, nuclear extracts and Electrophoretic Mobility Shift Assays (EMSAs)

HaCaT cells were grown as described above. Nuclear extracts from HaCaT cells were prepared by standard methods as described before[21]. The purification of His-ΔNp63α protein was performed according to standard protocols as described previously[13]. EMSAs were performed with either 100 ng of recombinant proteins or 5 μg of nuclear extracts and end-labeled double-stranded oligonucleotides as previously described[13]. Protein-DNA complexes were resolved by gel electrophoresis on 5% non-denaturing polyacrylamide gels in 1 × TBE buffer at room temperature. The gels were dried and visualized by autoradiography after electrophoresis. Anti-p63 antibodies used for supershift experiments have been described before[21].

### Plasmids

Recombinant plasmids were constructed using standard molecular biology protocols. ChIPed-DNA sequences corresponding to *NR3C1*, *ZNRF2*, *STAT6*, *HSF2*, *NOTCH2NL*, *MXD3*, *B4GALT1*, *AHR*, *YTHDF3*, and *FBXO32 *were PCR amplified and cloned into Kpn I – Nhe I or KpnI – Spe I sites of the pGL3-basic vector (Promega) containing the thymidine kinase (TK) minimal promoter[10]. All constructs were verified by sequencing.

### Transient transfections and reporter assays

Ptk2 cells were seeded in 6 well plates the day before transfection. Transfections were performed using Fugene 6 reagent (Roche) according to the manufacturer's protocol. One μg of each luciferase reporter construct was transfected per well along with 0.25 μg of pCMVLacZ plasmid to serve as an internal control for transfection efficiency. Reporter assays were performed as previously described[21]. Means and standard deviations were calculated based on data from three independent transfection experiments.

### siRNA knockdown of p63

HaCaT cells were seeded 24 hours prior to transfection in 100 mm plates. Transfections were performed with cells at 30–40% confluency with SiGenome SMART pool Human TP73L NM-003722 (Dharmacon) using Lipofectamine 2000 (Invitrogen). Cells were collected 48 and 60 hrs after transfection and analyzed for knockdown of p63 by Western blot analysis and quantitative PCR. For Western blot analysis, cell extracts were prepared by resuspending the pellets in Laemmli sample buffer (Bio-Rad). Approximately an equal amount of each sample was denatured at 96°C for 10 minutes. Blocking of the membrane was performed with 5% non-fat dry milk diluted in 150 mM NaCl, 10 mM Tris pH 7.5, and 0.1% Tween 20. Primary rabbit anti-p63 (RR14) was used for detection of ΔNp63 protein levels and membrane was stripped and re-probed with antibodies against β-tubulin to demonstrate equal loading. For quantitative PCR, RNA was extracted from siRNA-transfected and mock-transfected HaCaT cells and subsequently reverse transcribed. Quantitative PCR was performed with 2 μl of 1:5 diluted cDNA, 10 μM of each primer and the suggested amount of SYBR Green I dye (Bio-Rad) according to the manufacturer's instructions. Cycling parameters were as follows: 95°C for 8 minutes followed by 35 cycles at 95°C for 15 seconds and 60°C for 1 minute. Fluorescent data were specified for collection during the 60°C step. Data were normalized to the reference gene β-2-microglobulin. All reactions were repeated at least three times in triplicates and the relative expression level of each gene was determined.

## Authors' contributions

All experiments were carried out by BB and KO. BB, KO, and SS drafted the manuscript. All authors read and approved the final manuscript.

## Supplementary Material

Additional File 1 List of oligonucleotide sequences used for EMSA. p63 binding sites are indicated in bold. Click here for file

Additional File 2 Western Blot demonstrating the levels of ΔNp63α protein in mock transfected HaCaT cells and HaCaT cells transfected with p63 siRNA. Click here for file

Additional File 3 (A) List of oligonucleotide primers used for RT-PCR. (B) List of oligonucleotide primers utilized in the independent ChIP assays. Click here for file

## Figures and Tables

**Table 1:** List of known genes located within 100 kb of the DNA sequences isolated after ChIP that are putative targets of p63.

**p63 Binding Fragment**	**Chromosome Number**	**Nearest gene**	**Location**	**Description**	**Accession Number**	**Gene Ontology**
A2	3p12	SHQ1	3' end	SHQ1 homolog (S. cervisiae)	NM_018130	RNA processing
A4	7p15	AHR*	intron 10	Aryl hydrocarbon receptor	NM_001621	Transcription
A5	13q22	KLF12	intron 6	Kruppel-like factor 12	NM_0072498	Transcription
A6	11p15	ARNTL*	5'end	Ayrl hydrocarbon receptor nuclear translocator-like	NM_001178	Transcription
B2	11cenq22	ME3	intron 1	Malic enzyme 3	NM_001014811	Oxioreductase activity
B5	12q13	STAT6*	intron 19	Signal transducer and activator of transcription 6	NM_003153	Transcription
C3	7p15.1	ZNRF2*	intron 1	Zinc and ring finger 2	NM_147128	Ubiquitin ligase activity
C6	6q22.31	HSF2*	intron 10	Heat shock transcription factor 2	NM_004506	Transcription
C10	2q31-q32.1	CHN1	intron 5	Chimerin 1	NM_001025201	Signal transduction
C11	8q24.13	FBXO32*	intron 4	F-box protein 32	NM_058229	Protein degradation
D2	5q21.3	PJA2*	5' end	Praja2, RING-H2 motif containing	NM_014819	Ubiquitin ligase
D5	5q31.3	NR3C1*	intron 2	Nuclear receptor subfamily 3	NM_00176	Signal transduction
D7	Xq21.1	POF1B*	5' end	Premature ovarian failure	NM_024921	Cytoskeleton organization
D9	8q12.3	YTHDF3	3' end	YTH domain family member 3	NM_152758	Unknown
D12	6p21.3	DDR1*	intron 3	Discoidin domain receptor, family member 1	NM_013993	Cell adhesion
E2	10p12.1	GPR158*	intron 4	G-protein coupled receptor 158	NM_020752	Signal transduction
E6	5q31.1	SLC22A4	intron 1	Solute carrier family 22, member 4	NM_003059	Transport
E12	9q13	B4GALT1*	intron 1	UDP-Gal:betaGLCNAc beta 1,4-galtosyltransferase	NM_001497	Carbohydrate metabolism
G9	12q24.31	AACS	3'end	Acetoacetyl-CoA synthetase	NM_145248	Metabolism
H3	2q21.1	WASPIP*	intron 2	Wiskott-Aldrich syndrome protein interacting protein	NM_003387	Cytoskeleton organization
H4	15q21.2	SPPL2A	5' end	Signal peptide peptidase-like 2A	NM_032802	Protein degradation
H6	7p15-p14	TBX20	5' end	T-Box 20	NM_020417	Transcription
H8	12q13	SLC38A4	5' end	Solute carrier family 38, member 4	NM_018018	Transport
H12	3q27	MCCC1	intron 10	Methylcrotonoyl-Coenzyme A caboylase 1	NM_020166	Metabolism
I5	22q12.3	SYN3*	intron 6	Synapsin 3	NM_003490	Neurotransmitter secretion
J1	7p12-p11.2	GRB10	intron 3	Growth factor receptor-bound protein 10	NM_00100549	Signaling
J2	1q21.2	NOTCH2NL*	intron 1	Notch homolog 2	NM_203458	Signal transduction
J3	14q32.3	MARK3	intron 4	MAP-microtubule affinity regulating kinase 3	NM_002376	Kinase activity
J5	5q35.3	MXD3	intron 4	MAX dimerization protein 3	NM_031300	Transcription
J7	8q12.1	RGS20*	intron 4	Regulator of G-protein 20	NM_003702	Signal transduction
E1	3q25.3	TADA3L	3' end	Transcriptional adaptor 3	NM_006354	Transcription

Shown are known genes located closest to each p63 ChIPed DNA sequence and the corresponding chromosome number. Also shown are the location of the p63-binding site within each gene, NCBI accession number, and gene function.  Asterisks indicates those genes that were also identified by other studies.

**Table 2:** List of hypothetical genes that are putative target genes of p63 located within 100 kb of ChIPed DNA sequences.

**p63 Binding Fragment**	**Chromosome Number**	**Nearest Gene**	**Location**	**Description**	**Accession Number**	**Gene Ontology**
A12	10q22.2	C10orf11	intron 4	Chromosome 10 open reading frame 11	NM_032024	Unknown
B4	18q12.3	LOC284260	intron 7	Hypothetical protein	XM_211408	Unknown
C1	15q26.1	LOC441732	NL	Hypothetical LOC441732	ND	Unknown
D1	4q21.22	LOC441026	NL	Heterogenous nuclear ribonucleoprotein A3 pseudogene	NG_005584	Unknown
D10	1p32.3	C1orf34	NL	Chromosome 1 open reading frame 34	ND	Unknown
E8	5q31.1	LOC642961	3' end	Hypothetical protein	XM_931289	Unknown
F9	20q11.1	LOC645487	5' end	Hypothetical protein	XM_932932	Unknown
G12	3q13.13	LOC440973	NL	Similar to Nuclear transcription factor Y subunit beta	ND	Unknown
H11	5q13.2	LOC644420	intron 1	Hypothetical protein	XM_927568	Unknown
I2	9p11.2	FAM27E1	NL	Family with sequence similarity 27 member E1	NM_001024608	Unknown
I4	10q23.31	LOC643529	intron 3	Hypothetical protein	XM_931612	Unknown

Shown are hypothetical genes located closest to each p63 ChIPed DNA sequence and the corresponding chromosome number. Also shown are the location of the p63-binding site within each gene, NCBI accession number, and gene function. NL, Not localized; ND, Not determined.

**Table 3:** ChIPed DNA sequences that correspond to two potential target genes.

**p63 Binding Fragment**	**Chromosome Number**	**Nearest Gene**	**Location**	**Description**	**Accession Number**	**Gene Ontology**
A8(a)	19q13.32	ZNF541	3' end	Zinc finger protein 541	NM_032255	Transcription
A8(b)	19q13.32	NAPA*	5' end	N-ethylmaleimide-sensitive factor protein	NM_003827	Transport
B1(a)	6q23.3	FLJ46906	3' end	Hypothetical gene supported by Ak128874; BC071813	XM_928441	Unknown
B1(b)	6q23.3	CCDC28A	5' end	Coiled-coil domain containing 28A	NM_015439	Unknown
B3(a)	12q22	LOC642961	3' end	Hypothetical protein	XM_931709	Unknown
B3(b)	12q22	MRPL42	5' end	Mitochondrial ribosomal protein L42	NM_014050	Protein biosynthesis
C7(a)	20p12.1	WFDC12	NL	WAP four-disulfide core domain 12	NM_080869	Protease inhibitor
C7(b)	20p12.1	LOC613266	NL	Hypothetical LOC613266	ND	Unknown
D6(a)	1q32.1	Clorf116*	NL	Chromosome 1 open reading frame 116	NM_023938	Unknown
D6(b)	1q32.1	LOC653098	5' end	Similar to chromosome 1 open reading frame	XM_925981	Unknown
E10(a)	1p21-p13	CSF1	3' end	Colony stimulating factor 1	NM_000757	Cell proliferation/differentiation
E10(b)	1p13.2	AHCYL1	5' end	S-adenosyl homocystein hydrolase-like 1	NM_006621	Adenoyslhomocyteinase
G5(a)	21q22.11	LOC728598	5' end	Similar to oligodendrocyte transcription factor 1	XM_001127883	Transcription
G5(b)	21q22.11	LOC728409	3' end	Hypothetical protein	XM_001128368	Unknown
G10(a)	13q34	LOC122258	5' end	Hypothetical protein	NM_145248	Unknown
G10(b)	13q34	LOC400163	5' end	Hypothetical protein supported by BC034786	ND	Unknown
H1(a)	6q15	CYCSP16	NL	Cytochrome c, somatic pseudogene 16	NG_002966	Unknown
H1(b)	6q15	RNGTT	NL	RNA guanylytransferase and 5' phosphatase	NM_003800	mRNA processing
H5(a)	15q24	CRABP1	3' end	Cellular and retinoic acid binding protein 1	NM_004378	Signal transduction
H5(b)	15q25.1	IREB2	5' end	Iron-responsive element binding protein 2	NM_004136	Metabolism

Shown are the genes located closest to each p63 ChIPed DNA sequence and the corresponding chromosome number. Also shown are the location of the p63-binding site within each gene, NCBI accession number, and gene function. NL, Not localized; ND, Not determined; Asterisks indicates those genes that were also identified by other studies.
